# A Scenario-Based Hardware-in-the-Loop Platform for Real-Time Road-Load Emulation on Production Micromobility Drives

**DOI:** 10.3390/s26196320

**Published:** 2026-10-07

**Authors:** Assem Meghawer, Martin Võip, Mahmoud Ibrahim, Anton Rassõlkin

**Affiliations:** Department of Electrical Power Engineering and Mechatronics, Tallinn University of Technology, Ehitajate tee 5, 19086 Tallinn, Estonia; martin.voip@taltech.ee (M.V.); mahmoud.mohamed@taltech.ee (M.I.); anton.rassolkin@taltech.ee (A.R.)

**Keywords:** hardware-in-the-loop, road-load emulation, software-defined electric vehicles, e-scooter, test bench characterization, real-time control, efficiency map, sampling rate, micromobility, repeatability

## Abstract

Software-defined electric vehicles have reached micromobility, creating a growing need to evaluate how controller software shapes drive performance and energy use. This requires experimental platforms with quantified emulation fidelity and measurement performance. This paper presents a scenario-based hardware-in-the-loop platform combining a production e-scooter drive, a controlled magnetic particle brake, and a real-time road-load model. The platform reproduces rolling resistance, aerodynamic drag, and speed-gated inertial demand for a nominal 90 kg vehicle. Across eight repetitions of a scaled ECE-15 urban cycle, including a cold first run, the protective load cap retained 99.51% of the commanded load impulse. A post hoc sensitivity analysis of the seven warm runs gave 99.71%. Measured torque and speed tracking root-mean-square errors over all eight runs were 0.704 N·m and 0.593 km/h. Separate electrical characterization produced a machine efficiency map peaking at 86.3% and cycle-energy measurements with a coefficient of variation of 0.72%. Subsampling unfiltered 200 kHz recordings at every sampling offset gave a maximum absolute cycle-energy error of 0.14% at 2 kHz, a hundredfold reduction in retained samples under the tested conditions. These results provide a quantified experimental basis for micromobility drive testing and subsequent controller-software comparisons.

## 1. Introduction

Electric micromobility vehicles are now a routine part of urban traffic, and their drives are increasingly defined by software [1]: controller behavior is updated over the air [2], and a change shipped to a fleet can alter how much energy a vehicle uses without any physical modification. Evaluating such a change before it ships requires a bench that can put a production drive through a repeatable, representative load, and that can be trusted to report what it did.

Reproducing road load on a bench is established practice for passenger vehicles. There are standardized coast-down procedures for deriving road-load coefficients and setting a dynamometer to imitate the track [3,4], and the mature motor-in-the-loop literature for exercising electric powertrains without a vehicle exists [5,6]. Micromobility vehicles have not inherited this infrastructure. Their energy behavior is characterized mostly from instrumented road use [7,8,9], and where benches for this class do exist, they state their capabilities without quantifying how closely the delivered load follows the commanded one [10].

The distinction matters because a road-load emulator is itself an instrument, and an instrument that has not been characterized cannot support the claims made from it. A bench that commands a load profile does not necessarily deliver it. Actuator dynamics, saturation, and the torque available from the drive under test all intervene, and on a micromobility drive they intervene hard. The bench described here has rotating inertia equivalent to 0.54 kg of vehicle mass standing in for a vehicle of 90 kg, so almost all of the inertia has to be synthesized actively. The production drive also has far less torque headroom than a car-scale unit, so the platform carries a protective load cap, and the cap engages on every run. Neither condition holds on the car-scale benches where road-load emulation accuracy has been examined [11].

A second gap concerns the measurements taken from such a bench. The influence of acquisition bandwidth on measured electrical power is well studied at the level of waveforms and power-quality quantities [12,13,14]. The quantity actually quoted in consumption studies, however, is energy integrated over a cycle, and how far the acquisition rate can be reduced before that integral moves has not been established for this vehicle class.

By envelope we mean the bound on the load the platform can actually apply, which on this bench is set by a protective cap. This paper addresses two research questions:

RQ1. What fraction of the commanded load impulse passes the platform’s protective cap over a scaled standard cycle, and what bounds its envelope?

RQ2. How does the acquisition rate affect cycle-energy figures computed from full-rate electrical recordings on such a bench?

Our contributions are the following.

1. A characterized road-load emulation platform for a production micromobility drive: This is described together with its measured characterization (Section 3), including its real-time control link, the two operating modes it runs in, and why results from them are not interchangeable.
2. Cap-loss and tracking figures with confidence intervals over repeats (Section 5): The platform passes 99.51% (95% confidence interval (CI) [99.05, 99.97]) of the commanded load impulse through its protective cap over eight runs, a figure taken on the command path and reported beside the measured impulse and the measured tracking error. The cap withholds 0.49% while being active for 8.38% of the moving run, and most of what it withholds falls at the end of decelerations, not at launch. Only the impulse reading answers RQ1.
3. A machine efficiency map (250–520 rpm, 10–30% of load command): A by-product of the acquisition-master session, referenced to the machine terminals, peaking at 86.3% at 520 rpm, 30% load, with its repeatability measured at a repeated reference point (Section 6).
4. A sampling-rate bound for cycle energy (Section 6): Energy computed from these recordings changes by 0.14% or less down to 2 kHz, by 0.77% at 200 Hz and by 5.99% at 10 Hz, taking the worst case over every sampling phase and every run, because the errors change sign and cancel in any average.

Throughout, the accuracy figures quantify the fidelity to the load the platform’s own model commands, not agreement with a road vehicle; Section 4 makes that distinction precise.

## 2. Related Work

### 2.1. Software-Defined Vehicles and the Testing They Need

A software-defined vehicle is one whose functions are set by software on centralized computing platforms and can be changed after production, typically over the air, rather than fixed by the hardware [1]. The secure delivery of such updates has a literature of its own [2]. The control functions themselves are increasingly developed and tuned in simulation, from classical controllers to learned policies [15,16,17,18], so the evidence behind a new release is often simulation evidence. Less is written about the other end of the process: before a control change is released to a fleet, someone has to show on the production hardware that it does what it claims. For cars, this is carried out on the hardware-in-the-loop and motor-in-the-loop benches reviewed below. For a micromobility vehicle, whose drive is a low-cost hub motor with a closed production controller, the equivalent bench is the subject of this paper, and its usefulness for that purpose rests on knowing its own fidelity.

### 2.2. Energy Testing of Micromobility and Light Electric Vehicles

Measured energy consumption for micromobility, and for light electric vehicles more broadly, comes mostly from instrumented road use, not from benches. Fleet and field studies characterize shared e-scooters in service [7] and identify the real-world factors that dominate consumption for electric motorcycles [8]. On-board data acquisition during real driving has been used to profile two-wheeler powertrain behavior [9], and open datasets have been used to model micromobility energy use statistically [19]. A parallel line validates simulation models of light powertrains against measurement [20,21,22]. Bench-based work on this vehicle class is comparatively rare: the chassis-dynamometer energy balance of Racewicz and Kazimierczuk [23] is among the few, and dynamometer coast-down has been applied to e-scooter tires specifically [24].

Which cycle to run is a related question. Regulatory light-duty cycles are documented in their own right [25,26], and the choice of cycle is known to move computed consumption [27]. We found no established procedure for scaling such a cycle down to the speed range of a micromobility vehicle; work in this area constructs bespoke two-wheeler cycles from measured use [28]. Halving the speed ordinates while preserving the time base, as described in Section 4, is this work’s own choice. It keeps the cycle’s structure and brings it inside the vehicle’s speed range, and it is reported here as such.

### 2.3. Road-Load Emulation on Test Benches

For cars, road-load reproduction on a bench is codified in standards: coast-down measurement of road-load coefficients [3,29] is paired with their use to set dynamometer coefficients so that the bench imitates the track [4]. The reliability of such measurements on roller dynamometers has itself been examined [30], and one servo-dynamometer study reports a quantified discrepancy between bench and on-road energy for a route-emulating bench [31]. Heavier-vehicle load-simulation benches go further and publish explicit calibration procedures with steady-state and transient accuracy figures [32,33]. Recently, a configurable dynamometer for micromobility devices has been described, with road-load emulation and extensive instrumentation [10].

### 2.4. Hardware-in-the-Loop Powertrain Benches

Electric-motor-in-the-loop testing places a real machine inside a simulated vehicle so that calibration and control can be exercised without a vehicle [5,6]. The approach has been used to validate powertrain topologies [34] and to implement power-split strategies on a versatile bench [35], and the power hardware-in-the-loop literature more broadly has matured to the point of review [36]. Directly relevant here, Song et al. evaluate competing road-load simulation algorithms on a motor-in-the-loop bench [11], which is the closest methodological precedent to the present work. Every one of these benches, however, is at car, bus, tracked-vehicle, or grid scale.

### 2.5. Efficiency Mapping, Acquisition Rate, and Uncertainty

Efficiency maps and the loss mechanisms behind them are well covered for traction machines [37,38], including hub motors [39], with known sensitivity to how the map is produced [40,41]. The effect of acquisition bandwidth on measured electrical power is likewise established: signal bandwidth requirements for automotive drive power measurement [12], sampling-induced error in power measurement [13], sampling frequency effects on power-quality quantities [14], and aliasing when reading switched waveforms in hardware-in-the-loop settings [42]. Measurement uncertainty for powertrain benches has been treated formally [43], and the dynamic behavior of torque transducers, the reference channel on any such bench, has a dedicated metrology literature [44,45,46]. Digital-twin validation practice [47,48,49], including for electric-vehicle propulsion drives [50,51] and for e-bikes [52], separates the validation of the virtual model from the characterization of the physical asset; this paper does only the latter (Section 4). Open-source instrumentation for bench monitoring has also been described [53].

### 2.6. The Gap

Road-load emulation accuracy is reported for heavy- and passenger-vehicle benches [31,32], and the standards assume it [4], but the micromobility bench literature stops at describing capability. The micromobility dynamometer of Muthaiah et al. [10] specifies its road-load emulation and channel count without reporting delivered-versus-commanded accuracy over a cycle. The motor-in-the-loop benches that do evaluate road-load algorithms [11] operate at car scale, where rotating inertia is substantial and the drive has torque headroom; neither condition holds for a micromobility vehicle whose bench has almost no rotating mass and whose production drive has little torque headroom. Sampling-rate effects on measured power, meanwhile, are studied at the level of waveforms and power-quality quantities [12,13,14], but not, for this vehicle class, at the level of the integrated cycle energy that consumption figures are actually quoted from.

We therefore report what a micromobility hardware-in-the-loop bench delivers against what it commands, with confidence intervals over repeats; we characterize the machine over the range we sweep on the same platform; and we quantify how far the acquisition rate can be reduced before cycle energy is affected.

## 3. Platform Architecture and Instrumentation

### 3.1. Bench Configuration

**Figure 1:**
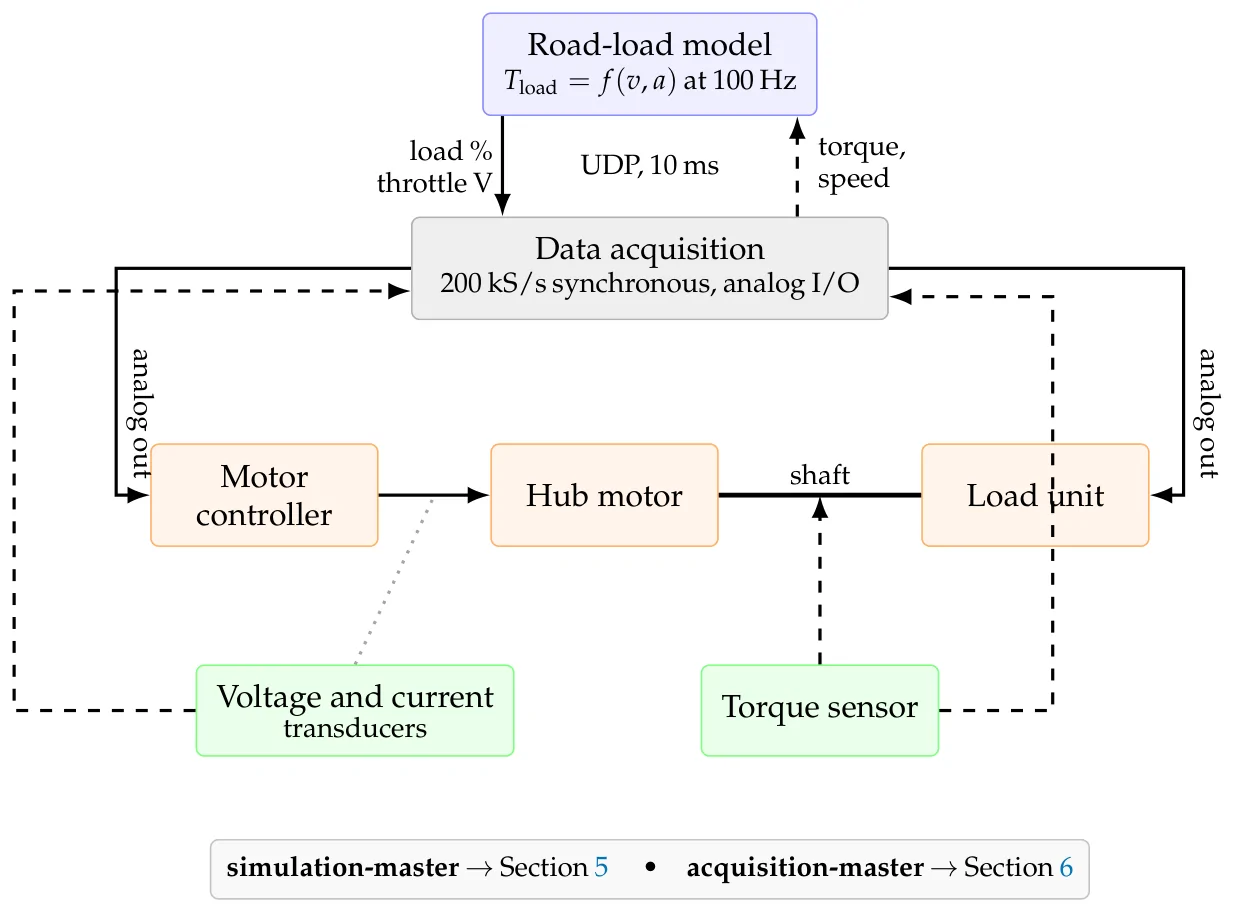
Platform architecture and signal flow. Solid arrows are commands, dashed arrows measurements. The thick solid line is the mechanical shaft; the grey dotted line locates the voltage and current measurements between the motor controller and hub motor.

**Figure 2:**
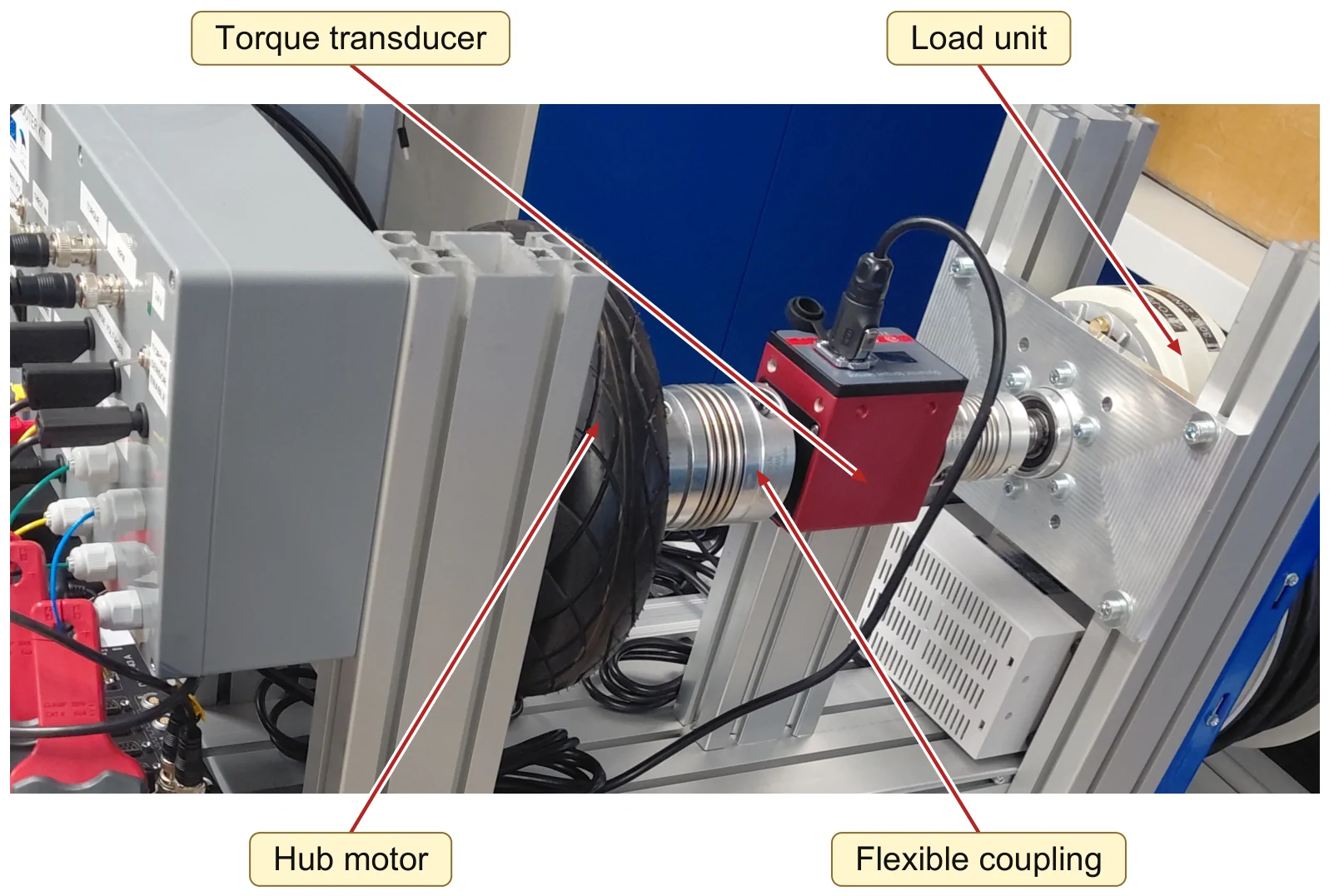
The drivetrain, viewed from the load-unit side: The hub motor drives through a flexible coupling into the in-line torque transducer and a second coupling to the load unit, all carried on an extruded frame. The transducer sits between motor and load, so what is measured is shaft torque delivered to the load.

The platform couples a production e-scooter hub motor to an electrically controlled load unit, a magnetic particle brake (ATO-PB-025, rated 25 N·m) driven by its current controller (KTC002, sold as a tension controller), through an in-line torque sensor (Figure 1 and Figure 2); Table 1 summarizes the measured characteristics and fitted models. The motor is driven by its own production controller, which accepts a throttle voltage over the range 0–4.74 V; the load controller sets the brake coil current from an analog command, expressed here as 0–100%. The brake nameplate specifies a maximum speed of 1800 rpm and coil ratings of 24 V DC, 1.24 A, 30 W; the controller’s rated output is 0–24 V DC, 4 A. Neither the motor controller nor the brake was modified or replaced, so the emulation acts on the same hardware and firmware a vehicle would carry on the road.

Two properties of this arrangement matter for what follows. The first is that the production motor controller is itself a speed controller. Measured open loop, road speed is linear in throttle, $v=1.783\;V_{\mathrm{th}}-0.305$ m/s, and it is held under load until the motor reaches its torque ceiling, at which point speed collapses abruptly instead of sagging away. The emulation does not need to close a throttle loop, because the drive hardware already closes one, and throttle voltage can be treated as a speed setpoint. The second is that the load side carries almost no rotating inertia. A coastdown measurement gives 0.0087 kg m^2^, equivalent to 0.54 kg of translating mass, against the tens of kilograms a vehicle would present. There is no flywheel, so any inertia the scenario calls for has to be synthesized by the load unit. Motor and load are consequently balanced against each other within tenths of a newton-meter at all times, and a net torque of a few hundredths of a newton-meter changes wheel speed measurably within a second.

The load unit is not a linear actuator, and the platform is designed around its measured behavior rather than a nominal specification. A magnetic particle brake transmits a torque that is nominally proportional to coil current, and the datasheet claims that proportionality from 5% of rated torque upward [54]. Measured, it has a command deadband of 4.75%, a minor-loop hysteresis of 0.16 N·m, and a gain that falls by about a third across the working range. Its static curve, the lookup that the feedforward and the cap both use, is calibrated from zero to 50% of command, where it reaches 11.8 N·m; the preflight check rejects any demand outside that range. The unit’s dynamics are dominated by transport delay: 0.36 s of pure dead time followed by a 0.16 s first-order lag, measured over 96 step edges across three load bands and across cold and warm runs, and this was found to be the same in all of them. The datasheet response of 0.12 s refers to the brake alone; the figures here include the controller and are what the loop sees. The split matters more than the combined response time, because dead-time phase grows without bound with frequency while lag phase saturates at 90°; the dead time sets the achievable loop bandwidth.

The load command is formed from two terms. A feedforward term inverts a measured lookup of the load unit’s static curve, so the deadband and the falling gain are compensated rather than left to the feedback. A discrete proportional–integral correction then acts on the error between the torque setpoint and the measured shaft torque, with anti-windup, at the 10 ms step of the loop; the gains used for every result in this paper are given in Section 5.1. The loop closes on the in-line transducer, which is also the reference channel for every torque figure reported (Section 7.3).

### 3.2. Signal Flow and the Real-Time Loop

Figure 1 shows the signal flow. The bench is a cyber-physical loop: a Simulink model of a scooter on a road computes the road-load torque the scenario demands and commands the bench to reproduce it in real time. The data acquisition system is the input/output layer between the two: it drives the motor controller and the load unit through analog outputs and returns shaft torque and wheel speed.

The model runs in Simulink on the acquisition host at a 10 ms fixed step, paced to wall-clock time by the simulation pacer; the loop is soft real-time, so timing is not deterministic, and link gaps are logged and screened against the criterion of Section 5.1. The loop runs over UDP (User Datagram Protocol). Each direction carries two double-precision fields: load command and throttle voltage outbound, torque and speed inbound. The measurement stream is nominally 100 Hz, but it arrives in bursts of roughly seven packets separated by tens of milliseconds of silence. A receiver that keeps only the newest packet at each step therefore sees fresh data several times less often than the nominal rate suggests, even for a channel that is fast at source. Draining the queue in order restores the full rate. In one verification run we measured 99.8 Hz with no starved steps; campaign-wide link statistics are given in Section 5.1. The speed resolution seen by the model improved twelvefold, from 0.06 m/s to 0.0049 m/s (0.018 km/h); this is the quantization of the value carried over the link, not the channel noise floor of Table 2.

### 3.3. Two Operating Modes

The platform runs in two distinct modes, and the results reported here come from both. In simulation-master mode the Simulink model closes the road-load loop in real time and logs the mechanical signals. This is the mode in which emulation accuracy (Section 5) is assessed. In acquisition-master mode the acquisition software plays a stored profile directly to the analog outputs, with no model in the loop, and stores every channel at full rate. This is the mode used for the electrical characterization of Section 6. The two are mutually exclusive on this installation because full-rate storage and the real-time transmission that carries the control link cannot both be active.

The separation bounds what may be compared across sections. The acquisition-master recordings reproduce the speed profile of the scenario but not the emulated road load, so energy figures computed from them characterize the drive at the operating points commanded and are not comparable with the simulation-master runs.

### 3.4. Measurement Channels

**Figure 3:**
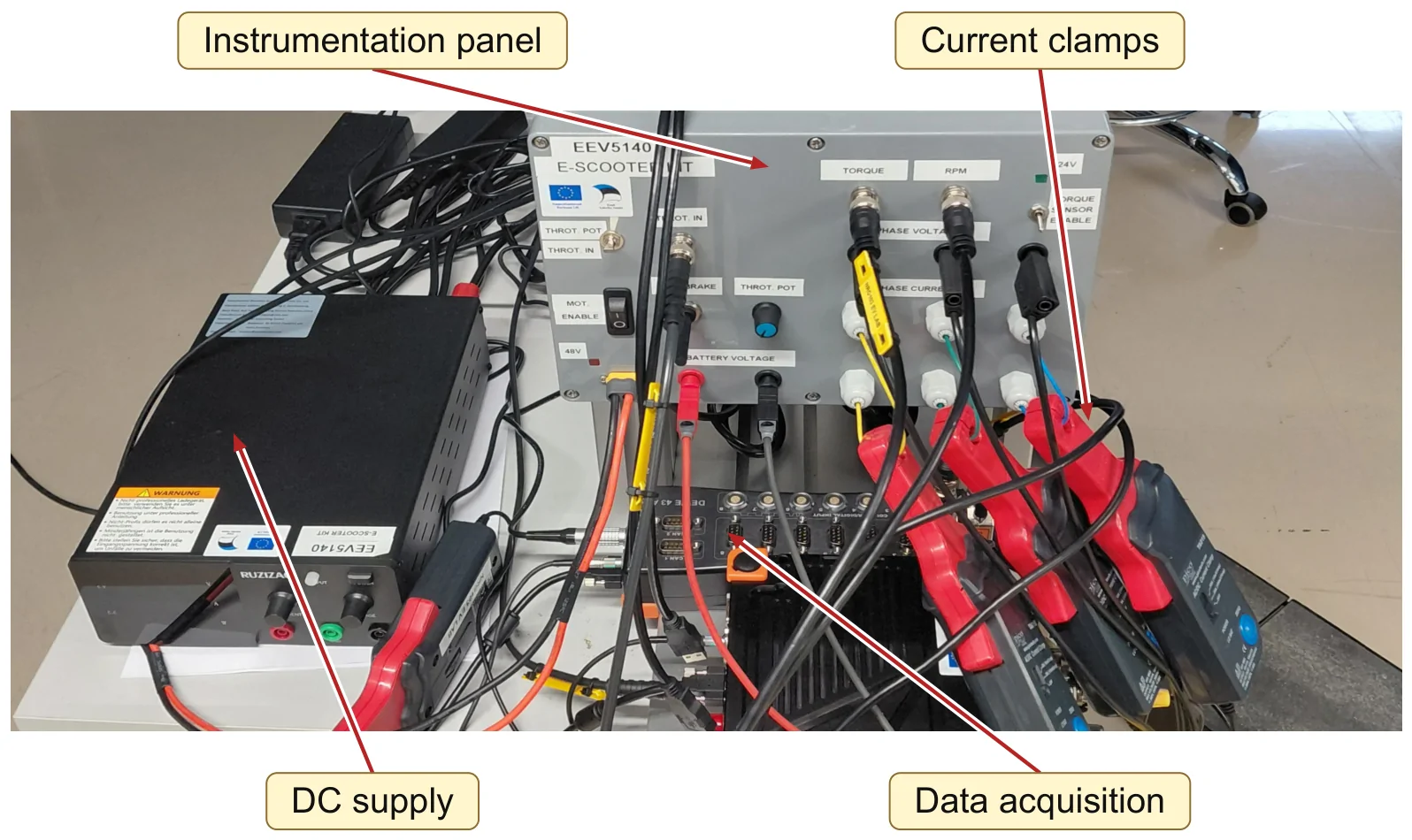
Instrumentation and acquisition. The instrumentation panel breaks out throttle, brake and enable lines together with the torque and speed channels; the clamp transducers feed the acquisition unit.

Nine analog channels are acquired synchronously at 200 kS/s: shaft torque, the tachometer signal, three phase voltages and three phase currents at the machine terminals, and the voltage of the 48 V supply. Figure 3 shows the instrumentation panel. Table 2 gives ranges and derivations. The in-line torque transducer is a DYN-200, rated for a 24 V DC supply. Torque is a direct 24-bit channel scaled at 6 N·m/V from the transducer’s ±5 V output, which is ±30 N·m full scale. The three phase voltages are measured line-to-line at the machine terminals, with the acquisition software’s power module set to its delta topology, which describes how the voltages are measured (three line-to-line, no accessible neutral) rather than the winding itself. The per-phase voltages and powers the module reports are calculated from those three measurements. The phase currents are measured with Pico TA018 AC/DC clamp transducers on their 60 A range at 100 A/V, specified to 20 kHz. Wheel speed is not measured as a voltage but computed from the pulse frequency of a 360-pulse-per-revolution encoder, with a trigger at 2.525 V and rearm at 1.082 V, and it is published asynchronously at approximately 500 Hz. Power quantities, both per-phase and total active power and the mechanical power formed from torque and angular velocity, are computed by the acquisition software’s power module from the synchronous inputs and are likewise asynchronous.

The distinction between synchronous and asynchronous channels affects how the exported data must be handled. Every channel is written onto the fastest channel’s time grid, and a slower channel with no new sample in a given row is written as zero, not held at its last value. A plain average over such a column is biased toward zero; at the worst-affected of the operating points reported in Section 6 (355 rpm, 30% load), the efficiency would read 3.3 percentage points low. All analysis in this work discards those rows explicitly.

### 3.5. Safety Interlocks and the Load Cap

Protection is applied in several layers, and the software limits are not relaxed between runs. The torque setpoint is saturated to 0–20 N·m and the load command is rate-limited to ±100%/s. A stall cutout reads unfiltered speed and declares a stall when throttle is applied and speed stays below 0.3 m/s for 0.3 s; it releases automatically after 1.0 s and latches permanently after three trips. It arms only once the wheel has exceeded 1.0 m/s so that a standing start is not mistaken for a stall.

The same block applies the protective load cap that Section 5 quantifies. At every step it evaluates the motor ceiling model of Section 4.3 at the present throttle voltage, takes 85% of that torque, converts it to a load command through the measured static curve, and clamps the command there. The load unit can therefore never be asked for more than the motor can hold at that throttle. The torque withheld by the clamp is logged sample by sample, so the cost of the protection is a measured quantity rather than a silent deviation.

A preflight check computes the entire scenario before anything is commanded, and it fails closed. It tests the demand against the setpoint saturation, the calibrated range of the load unit, and the measured motor ceiling model. A motor shortfall is reported as expected tracking degradation, not as a failure, because the platform is self-limiting: if the drive falls behind, the speed-dependent gate on the inertia term (Section 4.3) closes, the load falls, and the bench recovers. Every hardware run requires explicit operator confirmation; stop commands are issued on normal exit, on error, and on interrupt; and a physical emergency stop remains the primary protection.

## 4. Scenario and Road-Load Model

### 4.1. Drive Cycle

A scenario, in this paper, is a speed profile together with a road surface and a declared set of vehicle parameters. The speed profile is the ECE-15 urban driving cycle [55], taken from the regulation’s speed–time table as a piecewise-linear reference, with its speed ordinates scaled by one half and its time base preserved. The result is a 195 s profile of 16 breakpoints that peaks at 25.0 km/h. Scaling the ordinates instead of compressing time keeps the cycle’s three standing starts, its idle periods, and its acceleration durations intact; the accelerations are halved along with the speeds, and the cycle stays inside the speed range a micromobility vehicle actually occupies. The unscaled cycle peaks well above anything this class of vehicle reaches, so it cannot be used directly.

For this bench, the three standing starts constrain more than the peak speed does. Each demands the full inertia term from a stationary wheel, and in terms of the drive’s physical torque headroom, the platform comes closest to its limits during launch; Section 5.5 shows that the cap as implemented binds elsewhere.

### 4.2. Road-Load Model and Declared Parameters

The model computes longitudinal resistance from wheel speed and converts it to a wheel torque through the tire radius:

(1) $$T_{\mathrm{load}}=r\left[\;\left(mgC_{\mathrm{rr}}+\frac{1}{2}\rho C_{d}A_{f}v^{2}\right)\tanh\;\left(\frac{v}{v_{0}}\right)+g\left(v\right)\;m\;a\;\right],$$

with r= 0.127 m, m= 90 kg, $C_{\mathrm{rr}}=$ 0.013, ρ= 1.2 kg/m^3^, $C_{d}=$ 1.1 and $A_{f}=$ 0.45 m^2^. Here, *v* is the measured wheel speed, *a* is the inertia-term acceleration described in Section 4.3, which is taken from the reference profile rather than from the measurement, and g(v)∈[0,1] is the speed gate on that term, also described there. The steady terms are gated by a hyperbolic tangent with $v_{0}=$ 0.25 m/s so that a stationary wheel is not braked; without the gate, the bench demands rolling resistance at standstill, the controller integrates against it, and the next launch begins against an already-applied load. Here, *m* is the declared total mass of vehicle and rider. No rotating-mass factor is applied: the rotational inertia of the wheels is neglected, and the bench’s own rotating inertia, equivalent to 0.54 kg, is physically present on the shaft rather than emulated.

These are declared emulation parameters, not measurements of a physical vehicle. No instrumented scooter was weighed and no coastdown of a road vehicle was performed: the mass, the drag area, and the rolling coefficient define the vehicle the bench is asked to imitate. This distinction bears directly on what the accuracy results in Section 5 mean. They quantify how faithfully the platform reproduces the load its own model commands, which is a property of the emulator. Agreement with a real scooter on a real road is a separate question, one that needs a validation ride, so bench output is compared against the commanded signal. In digital-twin terms, the model is the virtual half and the bench the physical half; this paper characterizes the physical half, and the virtual half is declared rather than validated.

The rolling coefficient is chosen, not measured. The value 0.013 corresponds to dry asphalt and is selected from a surface menu in the model; the run script pins it and refuses to start if the selected surface differs from the campaign setting. Surface therefore cannot drift silently between runs, which matters because a change of surface moves the whole load curve without changing anything visible in the logged command.

**Figure 4:**
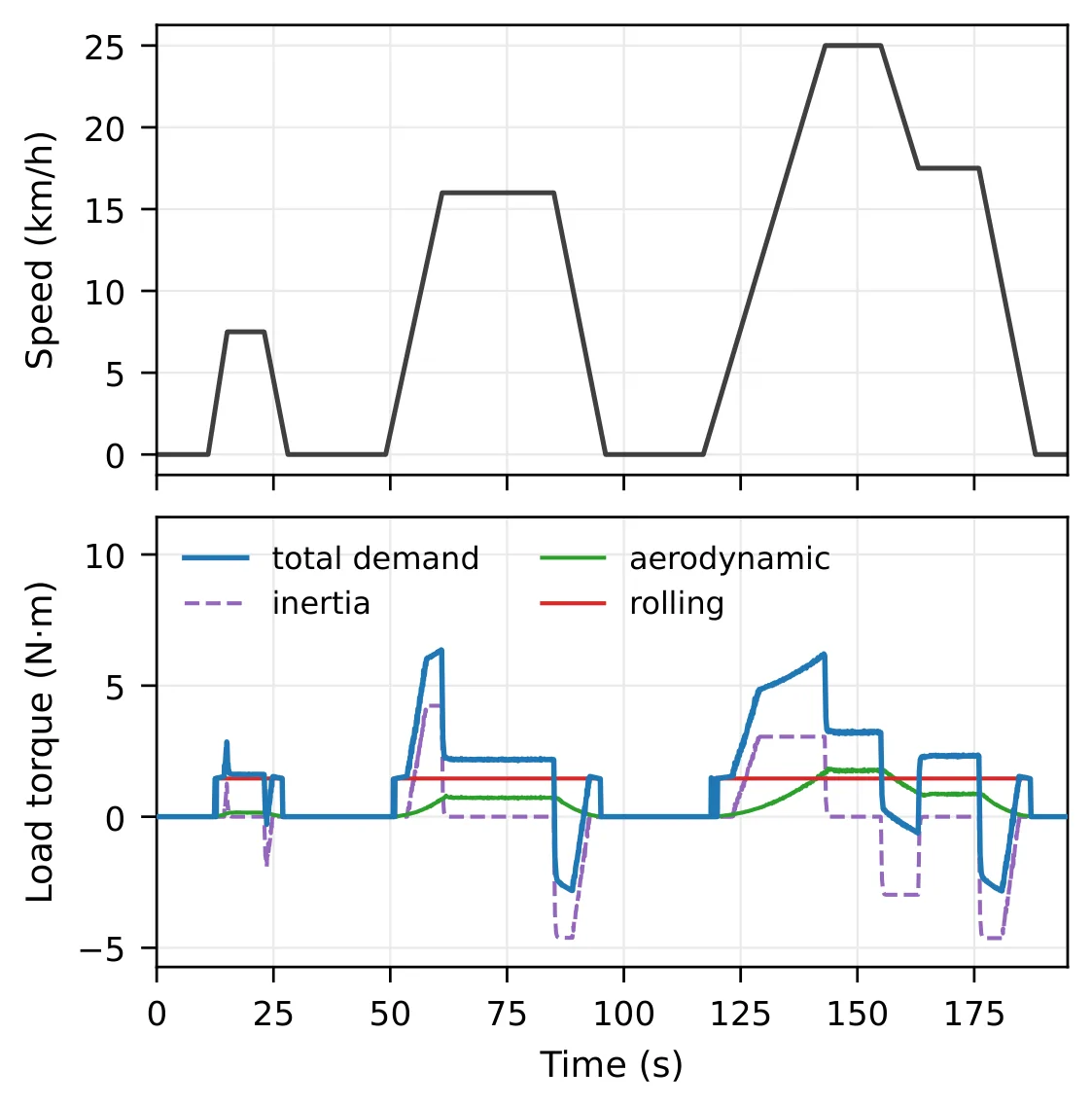
The scaled ECE-15 cycle (**top**) and the decomposition of the commanded load torque into rolling, aerodynamic, and inertia terms (**bottom**), computed from a logged run. The inertia term dominates every transient and is the only term that goes negative.

Figure 4 shows the cycle and the decomposition of the commanded load into its three terms, computed from a logged run rather than re-integrated from the speed breakpoints. Rolling resistance is constant above walking pace at 1.46 N·m. Aerodynamic drag grows as the square of speed, from roughly a third of the rolling term at half the cycle’s peak speed to 1.82 N·m at the peak, where it exceeds rolling resistance; the two together give a steady load of 3.28 N·m at peak speed. The inertia term dominates every transient and is the only term that goes negative.

### 4.3. Motor Ceiling as a Runtime Constraint

Equation (1) is unbounded, but the drive is not. The motor’s torque ceiling rises with throttle voltage as $4.64\;N\cdot m/V\;V_{\mathrm{th}}-0.64\;N\cdot m$, and throttle in turn sets road speed at 1.783 (m/s)/V, so the torque the bench can hold rises with the speed the profile is commanding at that moment. The demand is largest exactly where the available torque is smallest, at the start of each launch. Two design consequences follow.

First, the inertia term is a feedforward term with a speed gate. Its acceleration *a* is the derivative of the reference speed profile, smoothed by a 0.15 s first-order filter, multiplied by the gate g(v) of Equation (1), which is zero below 1.5 m/s of measured wheel speed, one above 3.0 m/s, and linear in between. Taking the acceleration from the reference rather than from the measured speed means that no measurement-derived acceleration feeds back into the load, so the loop that a measured acceleration would otherwise close through the load unit cannot form. The gate exists because, without it, the full inertia demand appears as soon as the reference begins to accelerate, while the throttle, and with it the motor’s ceiling, is still low. On a vehicle, or on a bench with a flywheel, a motor that cannot supply the inertia torque simply accelerates more slowly. On a bench whose rotating inertia is equivalent to only 0.54 kg of vehicle mass, the same shortfall is a braking load that would stop the wheel, so it has to be gated; that is a structural property of the bench, not a tuning fault. Second, the preflight check of Section 3.5 evaluates the whole cycle against this ceiling before any command is issued.

With the campaign parameters, the preflight calculation predicts a peak commanded load of 6.39 N·m over the cycle, and the load cap of Section 3.5 withholds a small fraction of the demanded impulse. Section 5 quantifies that fraction and shows where in the cycle it falls.

## 5. Emulation Accuracy

### 5.1. Run Design and Rejection Criteria

Accuracy was assessed over eight repeats of the scaled ECE-15 cycle in simulation-master mode, all at one controller setting ($K_{p}=0.47$, $K_{i}=5.30$, in load-command percent per newton-meter and per newton-meter-second), with 120 s of rest between runs. Thermal state was handled by procedure, with no warm-up run before the first repeat; the effect of that choice is quantified in Section 5.6. The repeat count was fixed in advance, and the resulting interval widths are reported as achieved.

Runs were screened against four rejection criteria: a control-link gap longer than 100 ms, speed tracking RMSE above 2 km/h, cap impulse loss above 5%, and any safety stop. All eight runs passed every criterion, and none was discarded, so the pooled figures below are over all eight runs. The link was sound throughout, with at most 5 dropped control-link samples per cycle (0.026% of samples) and a longest gap of 50 ms, well inside the criterion.

Two of these criteria were read differently from the plan written before the data were taken, and a third planned criterion was struck; the changes are recorded here rather than presented as the original protocol. The plan’s link-health rule had two clauses: a gap longer than 100 ms, and more than three dropouts per cycle. It did not say whether a dropout is a lost sample or a gap. Read as samples, four of the eight runs exceed three; read as gaps, none does, at most 3 per cycle. The gap clause, unchanged from the plan, was kept as the gate on physical grounds. A lost sample lasts one 10 ms step and the longest gap was 50 ms, short against the load unit’s 0.36 s dead time and 0.16 s lag, so it has no material effect on the delivered load. Both counts are reported. The plan’s cap rule read “cap binding above 5% of the moving run”. The cap engages on every run of this bench, for 8.38% of the moving run on average, so its active time cannot separate a sound run from a faulty one. The impulse it withholds can, and the plan’s own contribution statement pairs that quantity with impulse delivery, so the impulse reading was adopted. Both readings were settled after the runs had been seen. An ambient-temperature criterion was struck before the accuracy analysis because the bench has no temperature channel and it could never have been evaluated.

**Statistical treatment.** All intervals in this paper are Student-*t* 95% confidence intervals on the mean, reported as descriptive summaries of the repeats. On the pooled set of eight runs, every headline metric departs from normality (Shapiro–Wilk $p<10^{-3}$; $p<10^{-5}$ on all but the cap active time), because the sample is seven tight values plus one cold-bench outlier; pooled intervals should therefore be read as indicators of spread, not as intervals with exact coverage. For that reason, the medians and full ranges are reported alongside the following: impulse delivered 99.71% [98.15, 99.73], cap impulse loss 0.29% [0.27, 1.85], cap active time 7.99% [7.68, 10.93], tracking root-mean-square error (RMSE) 0.624 N·m [0.614, 1.265], and speed RMSE 0.505 km/h [0.481, 1.237]. The warm subset was formed by setting aside a run already seen to be extreme, so it is a post hoc sensitivity analysis and its intervals are descriptive in the same way.

### 5.2. Instrument Noise Floor

Interpreting a tracking error requires knowing what the instrument contributes. Table 2 lists the measured noise floors. Torque resolves to 0.00868 N·m at full bandwidth and the speed channel to 0.113 km/h at a steady low speed. The at-rest record cannot supply the latter, because a stationary wheel quantizes the speed channel to exactly zero; a dedicated full-bandwidth record at steady speed was needed to establish it. The speed floor is measured at one steady speed, and since the channel derives speed from pulse frequency, its quantization varies with speed; the figure is a resolution limit at that operating point, not a floor for the whole cycle.

In the acquisition-master session of Section 6, the torque channel read −0.0405 N·m at rest 23 min after it had been zeroed, roughly five times its own noise floor, so the channel is zeroed before every session instead of once per campaign. The fidelity session predates that practice, and its standstill samples serve as its zero reading: 0.00 N·m before the first launch of the session, and between −0.22 N·m and −0.14 N·m at every standstill from the first stop on, with no trend over the following 37 min (Section 5.5). With the wheel turning and nothing commanded, the channel read +0.034 N·m to +0.045 N·m in every run, steady within 0.012 N·m across the session. The tracking errors below are differences between a command and a reading from this channel, so the offset does not cancel in them. Its size is small beside the tracking errors themselves; for the absolute efficiency figures of Section 6, the offset measured in that session is carried forward.

### 5.3. Motor Ceiling Cross-Check

The preflight check and the load cap rely on a ceiling model that is linear in throttle voltage and was fitted at 1.0 and 2.0 V: $4.64\;N\cdot m/V\;V_{\mathrm{th}}-0.64\;N\cdot m$. It was verified against direct measurement at 2.5 V on three separate days: 10.78 N·m, 10.98 N·m, and 10.84 N·m, against 10.96 N·m predicted, a worst-case disagreement of 1.6%. We report the range and not a confidence interval, because the three measurements were taken on different days under uncontrolled thermal conditions and are not repeats of one quantity in the statistical sense. The ceiling is verifiable only at this throttle setting, because above it, the ceiling (13.3 N·m at 3 V) passes the top of the load unit’s calibrated static curve, 11.8 N·m at 50% of command, so the load unit cannot load the motor hard enough to make it droop within that range. Above 2.5 V the cap follows the fitted line, which the cycle reaches rarely, since the cap binds almost entirely at low throttle (Section 5.5).

### 5.4. What the Impulse Metrics Measure

Both impulse figures are ratios taken on the command path, so their definitions come first. Write $T_{\mathrm{set}}$ for the torque setpoint the controller receives, which is the road-load demand of Equation (1) after the saturation to 0–20 N·m, and $T_{\mathrm{short}}$ for the torque the protective cap withheld at the same instant. The cap acts on the load command, so $T_{\mathrm{short}}$ is obtained by mapping the command before and after the clamp through the load unit’s measured static curve and taking the difference. Integrating over the moving part of the run, defined as the samples with *v* above 0.05 m/s, gives Equation (2):

(2) $$I_{\mathrm{dem}}=\int T_{\mathrm{set}}\;dt,\;I_{\mathrm{short}}=\int T_{\mathrm{short}}\;dt,\;\mathrm{cap}\;\mathrm{impulse}\;\mathrm{loss}=\frac{I_{\mathrm{short}}}{I_{\mathrm{dem}}},$$

and impulse delivered is $1-I_{\mathrm{short}}/I_{\mathrm{dem}}$. The two are complements by construction. They are quoted together because a reader asking how much load was given up should not have to perform the subtraction, and the cap active time is quoted beside them because the same loss concentrated in a few seconds is a different claim from the same loss spread thinly. The negative excursions of Equation (1) enter neither integral because the setpoint is saturated first: the load unit brakes only, so the regenerative phases belong to the platform’s scope (Section 7.3) rather than to the impulse loss counted here.

These ratios quantify how much of the road-load demand survives the protective limiter, which is what RQ1 asks. They are not a comparison against the measured shaft torque. Agreement between the load the bench was told to apply and the load the transducer recorded is reported separately, as the torque tracking error and as a measured impulse (Section 5.5), and it is those figures rather than the impulse ratio that read through the instrument.

### 5.5. Answering RQ1

**Figure 5:**
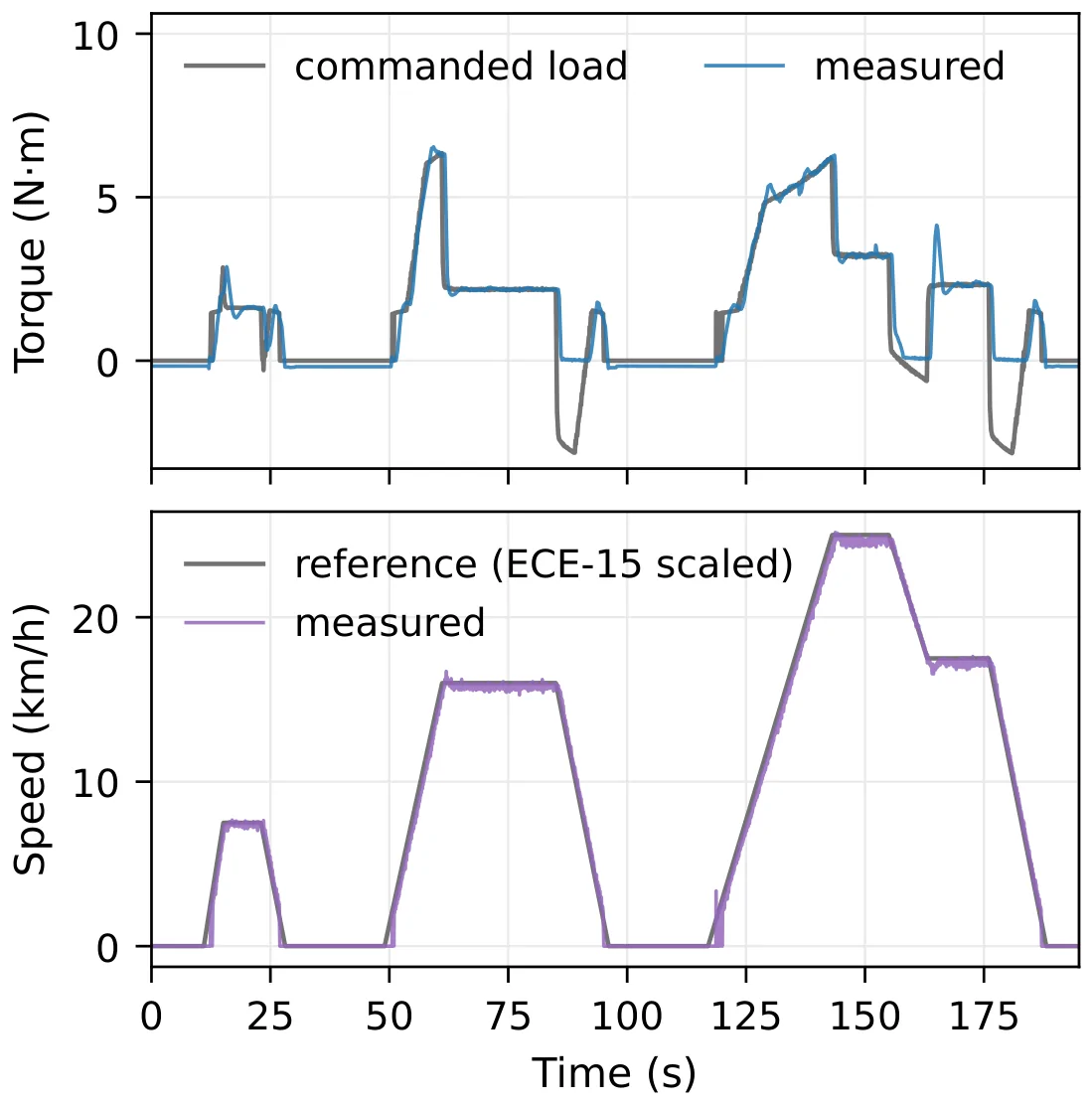
Commanded against measured load torque (**top**) and reference against measured speed (**bottom**) for one warm run. The commanded trace is the road-load demand before the saturation to 0–20 N·m; measured torque holds near zero where the command goes negative, because the platform emulates the traction side only.

**Figure 6:**
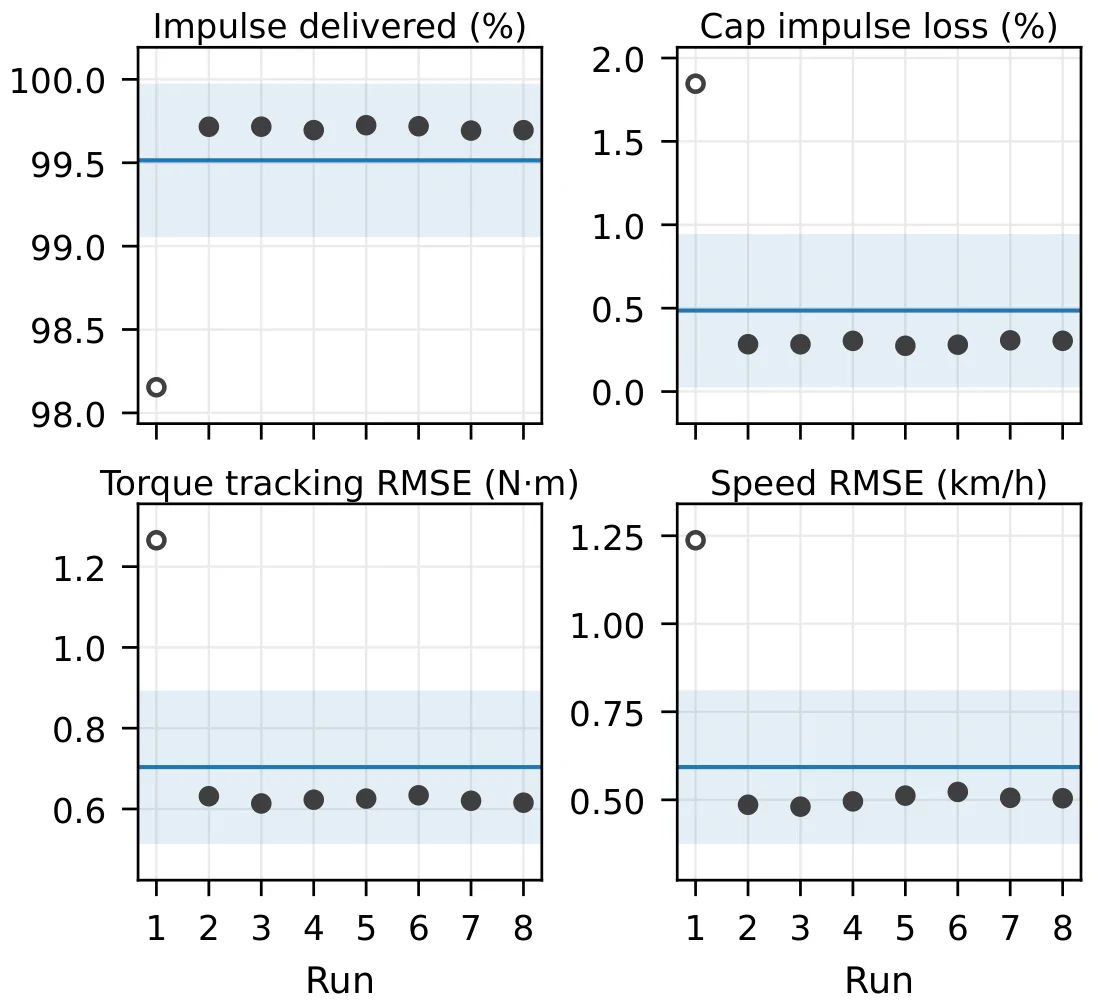
Accuracy metrics across the eight repeats. Filled markers denote the warm runs; the open marker denotes the first run of the session, taken on a cold bench. The blue horizontal line is the pooled mean, and the light-blue band is its 95% confidence interval.

Over eight repeats the platform passed 99.51% (95% CI [99.05, 99.97]) of the commanded load impulse through its protective cap, with 0.49% ([0.03, 0.95]) withheld. The denominator is the setpoint as issued: the road-load demand after the inertia gate and the saturation to 0–20 N·m. Both interventions are substantial. The gate holds the inertia term fully off for 12.05% of the moving run, and the saturation removes the negative excursions altogether, so the impulse figures are taken against the gated, non-negative command and not against the raw road-load equation; the gate is described in Section 4.3 and the traction-side scope in Section 7.3. Figure 5 shows commanded against measured torque and reference against measured speed for one warm run, and Table 3 collects the accuracy metrics, which Figure 6 shows run by run.

Two different quantities can both be called cap binding, and on this platform they differ by an order of magnitude. The cap was active for 8.38% ([7.50, 9.27]) of the moving run, but the impulse it withheld over that time was only 0.49%. Quoted alone, the active-time figure suggests a heavily capped emulation; the impulse figure alone hides how often the cap engages. We report both here, and we take the impulse reading as the cap-retention measure, since it is the quantity the research question asks about.

Where the cap binds is not where the design discussion of Section 4.3 would suggest. Classifying each withheld sample by the slope of the reference profile, 57–81% of the withheld impulse falls while the reference is decelerating and 12–43% while it is accelerating. The cold run is the upper case for decelerations and the lower case for accelerations; on the warm runs, the two shares sum to one, and on the cold run, the remainder falls where the reference is constant. In every run, 75–89% of the withheld impulse falls with the throttle command below 1 V. The reason is the cap’s law. The cap follows the throttle voltage. On the way down the throttle falls ahead of the wheel, and for the last seconds of every stop, the ceiling model falls toward zero while the setpoint still carries rolling resistance. The launches, where the motor’s shortfall is physical, account for the smaller part. They are nonetheless where the drive runs closest to its limit: over the inertia-gate ramp the setpoint reaches 85% of the cap, or about 73% of the modeled ceiling. The retention figure reported here is therefore set more by the cap’s own law than by the drive’s limits. A cap that also follows the measured speed would remove the binding at the end of stops. Such a cap takes the higher of two ceilings: the one at the present throttle, and the one at the throttle that would command the measured speed (Section 3.1). Replaying the eight logged runs through the cap block with that law brings the withheld impulse from 0.49% to 0.12% and the cap active time from 8.38% to 3.28%, and what remains falls at the launches. Speed alone would not suffice: at launch, the wheel lags the throttle, and a cap keyed only to speed binds more there. The replay holds the logged speed and load command fixed, so these are estimates; a re-run with the new law is the test.

Torque tracking RMSE, taken between the measured shaft torque and the setpoint $T_{\mathrm{set}}$ over the moving samples, was 0.704 N·m ([0.514, 0.893]). Because the setpoint is taken before the cap, this error includes the torque the cap withheld. Against the command that survived the cap, it is 0.700 N·m ([0.511, 0.889]), lower by 0.004 N·m, so the cap accounts for almost none of the error and the rest is the load unit’s own. Set against the steady load of 3.28 N·m at peak speed, Figure 5 shows where the error comes from: the transients, where the demand changes faster than the load unit’s 0.36 s dead time lets it follow, while on a settled plateau, the measured torque sits on the command. Over the settled part of the 16 km/h plateau (65–85 s of the cycle), the RMSE is 0.039 N·m pooled and 0.029 N·m on the warm runs, about a twentieth of the whole-run figure. Speed tracking RMSE against the reference profile, taken over the whole cycle, was 0.593 km/h ([0.375, 0.811]), a factor of 5.2 above the measured speed noise floor; on the warm runs alone, it is 0.501 km/h, a factor of 4.4. The whole-cycle figure includes the idles, where both speeds are zero; over the moving samples alone, it is 0.629 km/h pooled and 0.517 km/h warm, so the idles dilute it by little. Either way, it stands well clear of the instrument: it is the drive’s real tracking error under the emulated load, not an artifact of the channel. The speed noise floor itself comes from a steady low-speed record in acquisition-master mode, for the reason given above, and it is quoted here only as a resolution limit for the channel. Averaged over the repeats, the peak of the setpoint reached 6.359 N·m against 6.39 N·m predicted independently by the feasibility calculation, a cross-check of the road-load path that does not depend on the accuracy metrics themselves. The load command never reached its upper rail, and no stall protection trip occurred in any run.

Taken from the transducer rather than the command path, the measured impulse $\int T_{\mathrm{meas}}\;dt/I_{\mathrm{dem}}$ over the same moving samples was 103.16% ([100.56, 105.76]) pooled and 102.06% ([101.81, 102.32]) on the warm runs. The excess is dynamic. On the 16 km/h plateau, the mean reading agrees with the setpoint to within 0.004 N·m on every warm run. The surplus comes mainly from load removal: when the setpoint falls, the load unit releases later than it is commanded to. On the warm runs, about three quarters of the surplus accumulates while the setpoint is already at zero and the wheel is still turning. Because the load unit brakes only, the setpoint cannot go below zero, so the loop has no means of canceling that tail. The surplus is largest on the cold run, where the transients are longest. The measured path also carries the channel’s zero. At standstill with nothing commanded, the torque channel read 0.00 N·m before the first launch of the session and between −0.22 N·m and −0.14 N·m after every stop from the first one on, varying from one stop to the next. With the wheel turning and nothing commanded, it read a steady +0.034 N·m to +0.045 N·m in every run. A channel offset would not vary from stop to stop, so at least part of the standstill reading is a residual preload held in the couplings, which releases once the wheel turns. The logs do not show whether the rest is a zero offset of the channel. If all of it were, the shaft torque actually applied would sit above the setpoint by the same amount throughout the run, about 8% of the mean setpoint (2.32 N·m), because the loop regulates the reading rather than the torque. The torque RMSE would then be 0.743 N·m pooled and 0.663 N·m warm instead of 0.704 N·m and 0.623 N·m. A zero check at the start of each session, before the first launch, which the protocol now applies (Section 5.2), removes any channel offset present at the start of the session from future runs. The command-path ratio remains the figure quoted for RQ1, because it carries neither effect.

### 5.6. The Cold First Run

The first run of the day is an outlier on every metric: it recorded 98.15% impulse delivered, 1.85% cap loss, 1.265 N·m tracking RMSE, and 1.237 km/h speed RMSE, against roughly 99.71% and 0.623 N·m for the remaining seven; its measured impulse was 110.83% against 102.06% warm. On the cold bench the share of the withheld impulse falling in decelerations rises to 81%, yet on the 16 km/h plateau, the reading still sits on the setpoint, to within 0.008 N·m, so the whole cold penalty is in the transients. The run passed every rejection criterion and is retained.

The statistical treatment of Section 5.1 accounts for it: pooled intervals are read as spread indicators with medians and ranges beside them, and the warm subset is reported as a post hoc sensitivity analysis. Excluding the cold run, impulse delivery is 99.71% ([99.70, 99.72]) with a coefficient of variation of 0.01%, tracking RMSE 0.623 N·m and speed RMSE 0.501 km/h. Both figures are stated: the warm subset describes the instrument once it has settled, and the pooled figure describes a campaign of eight runs that includes a cold start. A warm-up cycle, run and discarded unlogged, has since been added to the protocol.

## 6. Machine Efficiency and Acquisition Rate

The measurements in this section were taken in acquisition-master mode (Section 3.3), where every channel is stored at 200 kS/s and the acquisition system plays the profile directly. Because the road-load loop is not running, these recordings characterize the drive at commanded operating points rather than the emulated cycle. All electrical quantities in this section are the three-phase quantities at the machine terminals, so the efficiencies reported are machine efficiencies, from three-phase electrical input to mechanical output; the inverter stage is outside the scope of this characterization.

### 6.1. Machine Efficiency Map

**Figure 7:**
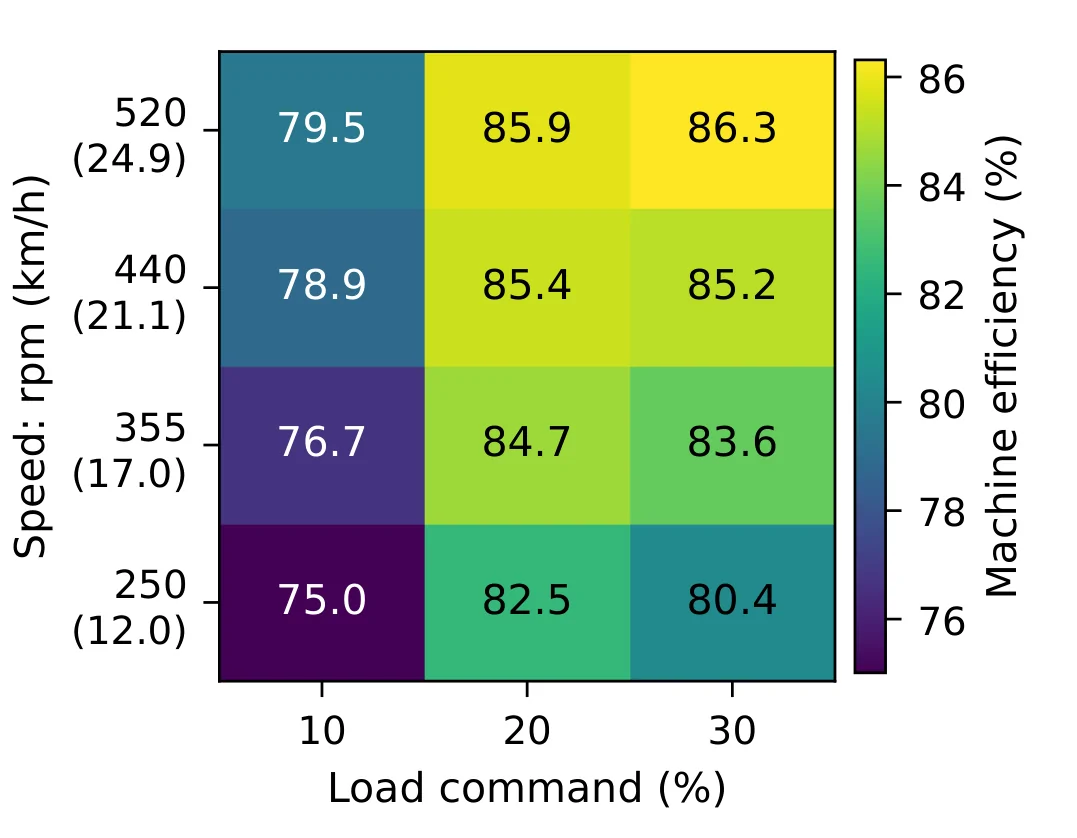
Machine efficiency, three-phase electrical input to mechanical output, over a grid of steady operating points spanning 250–520 rpm and 10–30% of load command, each held for 49–64 s (15 acquisitions in total; the repeated cell is the mean of its 4 repeats). Each value is the ratio of mean mechanical power to mean three-phase electrical power over the averaging window, referenced to the machine terminals; the inverter stage is not included.

The sweep held four speeds by four load levels, 19 acquisitions in all once the repeated cell is counted, each held for 49–64 s and averaged over the last ten seconds of the hold. Efficiency at each point is the ratio of mean mechanical power, formed from measured torque and angular velocity, to mean three-phase active power as the acquisition software’s power module reports it, both over that same ten-second window, so the ratio of mean powers is an energy ratio. The alternative, averaging per-sample efficiency, reads higher at light load because the ratio is taken before the averaging, by up to 0.14 percentage points over the cells shown. Figure 7 shows the map.

Efficiency peaks at 86.3% at 520 rpm, 30% load and exceeds 80% at the two highest load levels at every speed tested. The sweep covers 250–520 rpm and 0–30% of load command, and the map shows its loaded cells, 10–30%. At zero commanded load the torque reading is about 0.2 N·m and the torque-zero offset amounts to 18–22% of it, so an efficiency formed there is not quantitatively meaningful, and those four acquisitions are left out of the map.

Load levels were interleaved rather than swept monotonically, and one operating point was repeated four times across the session, so that variation within the session could be measured. Over 18 min the efficiency at that point spanned 0.52 percentage points. The spread is not monotonic in time, so it is not drift in the usual sense: it is the repeatability of the load unit’s torque at a fixed command, which over the same repeats spanned 13.3%. Efficiency, being a ratio of two quantities that move together, is far more repeatable than the operating point at which it is measured.

That figure is the observed range across four repeats, not a statistical bound and not an accuracy bound. The largest systematic identified is the torque channel’s zero offset, −0.0405 N·m measured in this session, which is 1.0% of the measured torque at the reference point and corresponds to about 0.8 percentage points of efficiency. Across the loaded cells of the map the same offset corresponds to 0.4–0.5 percentage points at 30% load, 0.7–0.9 at 20% and 1.6–2.2 at 10%. It is not the only one: the gain accuracies of the current and voltage transducers, and of the torque channel itself, enter the ratio directly and are taken from their factory specifications (Section 7.3). The map is therefore given to a tenth of a point, matching the repeatability figure, with its absolute accuracy bounded by those systematics.

### 6.2. Acquisition Rate and Cycle Energy

The sampling-rate study used three dynamic recordings of the cycle speed profile at a fixed 20% load command. Instantaneous three-phase power was formed sample by sample from the recorded channels rather than taken from the power module’s cycle-averaged output. The three voltage channels are line-to-line (Section 3.4), so phase quantities are reconstructed from them in the same way the power module does in its delta topology, $u_{1}=\left(u_{12}-u_{31}\right)/3$ and cyclically, giving Equation (3):

(3) $$p=\frac{1}{3}\left[\left(u_{12}-u_{31}\right)\;i_{1}+\left(u_{23}-u_{12}\right)\;i_{2}+\left(u_{31}-u_{23}\right)\;i_{3}\right],$$

with $u_{12},u_{23},u_{31}$ the three recorded voltages and $i_{1},i_{2},i_{3}$ the phase currents in the same sample. On a three-wire connection, this agrees with each of the three two-wattmeter forms to within 0.5%, and at a steady point on the cycle, it returns the machine efficiency the map of Section 6.1 gives at the same operating point. Power was integrated at the full 200 kHz rate (the 200 kS/s per-channel acquisition) over one common 195 s window in each recording, opened 11 s before the first non-zero speed command so that the cycle’s opening idle is included and the recording’s pre-roll and post-roll are not, to give a reference energy, then re-integrated using every *N*th sample to simulate a slower acquisition. The error of such a subsampling depends on where the retained samples fall on the switching carrier, so every one of the *N* possible sampling phases was evaluated for each factor (100 at 2 kHz, 20 000 at 10 Hz), and two rates that are not integer submultiples of the carrier, 1.7 kHz and 600 Hz, were added to the decades. Cycle energy at the reference rate was 4.825 Wh (95% interval [4.739, 4.911], descriptive), a CoV of 0.72% across the three runs.

**Figure 8:**
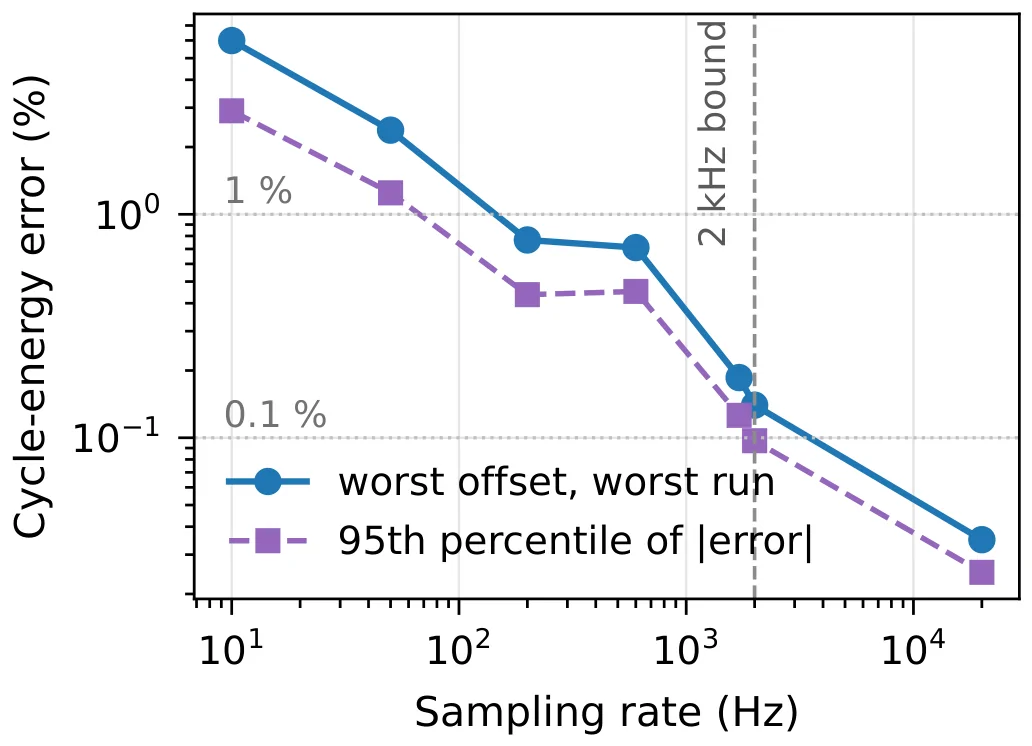
Cycle-energy error against simulated acquisition rate, relative to the 200 kHz reference, over every sampling phase of three runs: the worst case and the 95th percentile of the absolute error. The median is within 0.04% of zero at every rate because the errors take both signs.

Figure 8 shows the error against rate. The operative figure is the worst case over all sampling phases and all three runs; the median error is within 0.04% of zero at every rate and says nothing about a single recording. Down to 2 kHz the worst case is within 0.14% of the reference (95th percentile of the absolute error 0.10%) and 0.19% at the non-submultiple 1.7 kHz. Below that the worst case grows quickly: 0.71% at 600 Hz, 0.77% at 200 Hz, 2.38% at 50 Hz, and 5.99% at 10 Hz. In practical terms, energy figures subsampled from these recordings, for this drive at this operating point, are safe to about 2 kHz, where the worst case is well under the run-to-run variation of the energy itself (0.72%). At a few hundred hertz, the worst case matches that variation, and below it exceeds it.

Three cautions go with that number. The first concerns which statistic to quote. The errors take both signs, set by where the retained samples fall on the carrier, and cancel across phases and runs: at 10 Hz the median over all phases is +0.04% while the worst case is 5.99% and the 95th percentile 2.91%. A mean or a median would understate the risk to anyone making one measurement at one sampling phase, so we quote the worst case throughout. A check of the same recordings at one sampling phase gave 0.06% at 2 kHz, less than half the worst phase, so the sampling phase has to be covered. The second concerns method. The inverter switches at 26 kHz, measured from the phase-voltage spectrum of these recordings, with its second harmonic at 52 kHz; both lie below the 100 kHz Nyquist limit of the full-rate acquisition, so the full-rate record resolves the switching content present in these recordings, the carrier and its second harmonic. Every simulated rate below the second harmonic folds the carrier into the band. The current clamps are specified to 20 kHz, below the carrier, so carrier content in the current channels reaches the acquisition already attenuated and the aliasing seen here is, if anything, less than an unattenuated measurement would show. These rates were simulated by software subsampling of each full-rate recording, with no anti-alias filtering ahead of the decimation, whereas hardware acquiring at a low rate would filter first. The rates tested are also close to integer submultiples of the carrier: 26 kHz divided by 2 kHz is thirteen, and the lower rates divide it likewise, so at every rate from 2 kHz down the carrier and its second harmonic fold to within a few tens of hertz of zero frequency. An alias far from zero averages out over the cycle, while one that lands that close to zero survives the integration, with a sign set by where the samples fall on the carrier. That is why the error changes sign with sampling phase. With respect to aliasing, the figures here are therefore a worst case. A low-rate acquisition that filters before sampling differs in kind. It folds less switching content into the integral, but filtering the voltage and current channels separately discards any real power carried above the filter’s cutoff. That loss is one-sided and does not average out over sampling phase, whereas the aliasing error measured here takes both signs. The third concerns generality. All three recordings share one load level and one speed profile, and aliasing depends on the operating point and on the inverter’s switching content. The bound is established for this drive at this operating point. It should be re-established if the switching frequency, the load level, or the speed range changes materially, if transducers of a different bandwidth are used, or if the acquisition filters before sampling.

### 6.3. Answering RQ2

Unfiltered subsampling of the full-rate recordings moves computed cycle energy by 0.14% or less down to 2 kHz, by 0.77% at 200 Hz and by 5.99% at 10 Hz, with the caveat that a single measurement should be judged against the worst case over sampling phase and run, not the median. The bound has a practical consequence for the platform itself: an electrical channel at about 2 kHz could share the acquisition host with the real-time control link, which the full-rate storage of this section occupies on its own (Section 3.3). That would put energy under emulated load within reach of the same installation (Section 7.3). Such a channel filters before it samples, so it has to be checked against a full-rate record before it is relied on.

## 7. Discussion

### 7.1. Answers to the Research Questions

RQ1 is answered quantitatively. Over eight repeats the platform passed 99.51% (95% CI [99.05, 99.97]) of the commanded load impulse through its protective cap, and the measured torque tracking error against the setpoint was 0.704 N·m, concentrated in the transients: on a steady plateau, the reading sits on the setpoint to within 0.004 N·m. The envelope is bounded by the load cap, which withheld 0.49% of that impulse while being active for 8.38% of the moving run. Most of that loss falls at the end of decelerations, where the cap’s throttle-following law, not the drive, sets the limit. Once warm the repeatability is considerably tighter, at a coefficient of variation of 0.01% over seven runs. Which figure applies depends on one choice. For a campaign that begins with the warm-up cycle now in the protocol, the warm figure, 99.71%, is the relevant one. Without a warm-up, the first run can be expected to fall below it, as the cold run here did, and the pooled figure, 99.51%, shows the size of that effect over eight runs.

RQ2 is likewise answered quantitatively: computed cycle energy changes by 0.14% or less down to 2 kHz, by 0.77% at 200 Hz and by 5.99% at 10 Hz, taking the worst case over every sampling phase and run as the operative figure. The same acquisition session yields the machine efficiency map of Section 6, peaking at 86.3%.

### 7.2. Transferability

Five findings generalize beyond this bench. The first is that the distinction between how long a protective cap is active and how much it actually withholds matters. On this platform the two figures differ by an order of magnitude. A bench reporting only the first would appear heavily compromised. One reporting only the second would hide that the cap engages on every run. Any emulator with a protective limit should report both.

The second is that where a cap binds is a separate question from how much it withholds, and the answer can run against the design intent. This cap was built to protect launches, where the motor’s shortfall is physical, but on the bench, it binds mostly at the end of stops, because it follows the throttle command, and on the way down, the throttle falls ahead of the wheel. A limit keyed to a command signal inherits that signal’s timing; a limit that also follows the measured state does not.

The third is that a ratio can be far more repeatable than the operating point at which it is measured. Efficiency at a repeated reference condition varied by 0.52 percentage points across the session while the load unit’s torque at that same command varied by 13.3%. The two powers moved together: over the same repeats the mechanical output spanned 13.3% and the electrical input 13.0%, so their ratio spanned only 0.6%. A bench that quotes its commanded set point overstates how precisely it has placed the machine, and such a spread is a repeatability figure rather than an accuracy bound.

The fourth is that when a decimation error changes sign with sampling phase, an average understates the risk to a single measurement. At the lowest rate tested, the median error over every phase and run was +0.04% while the worst case was 5.99%, and at 2 kHz, a single-phase check gave 0.06% where the worst phase gives 0.14%. Sampling-rate guidance derived from averaged repeats, or from one sampling phase, is optimistic for anyone who will take one recording.

The fifth concerns what such figures are for. On a software-defined drive a controller update is judged by whether it moves the energy or tracking figures by more than the bench itself varies, and the repeatability figures established here give that scale. Run to run on the warm bench, the impulse coefficient of variation is 0.01% and that of the speed tracking RMSE is 2.92%. Efficiency at a fixed point repeats to 0.52 percentage points and cycle energy to 0.72% (CoV) over three recordings. All are within-session figures, and cycle energy is the one an energy claim rests on. They lead to a simple decision rule. Two controller versions are compared within one session, on a warm bench, with repeated runs of each. Both run against the same declared vehicle, so the comparison is like for like whether or not those parameters match a physical scooter. A difference between single runs that is smaller than the spread given here is not evidence of a change. Resolving a smaller difference needs repeats: the uncertainty of a mean falls with the square root of the number of runs, so the spread given here lets a user size the comparison before running it. What a given comparison resolves, and with what confidence, is then established by its own repeats. Day-to-day reproducibility is the subject of further work (Section 7.3).

### 7.3. Scope of the Results

**Emulated vehicle parameters.** The mass, drag area, and rolling coefficient in Equation (1) are declared values that define the vehicle the bench imitates, chosen rather than derived from a physical scooter. The accuracy figures therefore describe fidelity to the commanded load; agreement with a road vehicle is a separate question and would need a validation ride.

**Traction-side emulation.** The load unit brakes only, and the setpoint is saturated at zero, so what the platform applies during a deceleration is the non-negative part of the total demand, as Figure 5 shows: where the negative inertia term outweighs rolling resistance and drag, the applied load is zero, and the resistive terms are carried only while the total stays positive.

**Thermal state.** Thermal state is controlled by procedure rather than measured: a fixed rest between runs, and, since this campaign, a discarded warm-up cycle. Its effect is quantified in the retained cold first run (Section 5.6).

**Efficiency map scope.** The map covers 250–520 rpm and 10–30% of load command and is referenced to the machine terminals. Its repeatability is distinct from its accuracy: the largest systematic identified is the torque-zero offset, about 0.8 percentage points at the reference point, and the transducer gain accuracies that also enter the ratio are taken from factory specifications. The torque transducer’s class bound, 0.15 N·m (below), sets a wider limit: about 3 percentage points of efficiency at the reference point and under 2 at the peak, growing toward light load as the torque falls.

**Motor ceiling.** The ceiling model is verified at 2.5 V, the highest throttle at which the load unit’s calibrated static curve (11.8 N·m) can load the motor far enough to make it droop; above that setting, the cap follows the fitted line.

**Torque reference.** Every torque quantity is referenced to the single in-line transducer, which is also the sensor the control loop closes on, and its absolute values rest on the factory calibration: the datasheet for this transducer family bounds nonlinearity and hysteresis at 0.5% of full scale [56], 0.15 N·m here. Within the fidelity session the standstill reading was 0.00 N·m before the first launch and −0.22 N·m to −0.14 N·m after every stop (Section 5.5). The channel is now zeroed at the start of every session, before the first launch (Section 5.2), which holds any channel offset to the drift within one session.

**Cap law.** The impulse figures are specific to the cap as implemented, 85% of the throttle-derived ceiling; a cap that also follows measured speed is estimated to withhold 0.12% (Section 5.5).

**Scope of the electrical results.** The electrical channels are stored in acquisition-master mode, so the efficiency map and the sampling-rate bound characterize the drive at commanded operating points rather than under emulated road load (Section 3.3). Since unfiltered subsampling to 2 kHz moves cycle energy by 0.14% at most (Section 6), a low-rate electrical channel on the real-time path is the candidate for extending them to the emulated cycle.

**One drive unit, one session.** The results come from one motor, one load unit and one torque sensor, with the eight fidelity repeats taken in a single session; the motor-ceiling cross-check spans several days. Unit-to-unit variation and day-to-day reproducibility are the subject of further work.

**Sampling-rate bound scope.** The decimation study covers three recordings at one load level and one speed profile, subsampled without an anti-alias filter, so its errors are a worst case with respect to aliasing and the bound applies to this operating point and to unfiltered subsampling (Section 6.2).

## 8. Conclusions

A production micromobility drive can be exercised on a road-load-emulating bench with both paths quantified. On the command path, 99.51% of the commanded load impulse passes the protective cap over eight runs that include a cold start and 99.71% on the warm runs. At the shaft the measured tracking error against the setpoint is 0.704 N·m. The bound on the envelope is measured rather than assumed, and the cost of the cap is located in the cycle as well as counted. The same platform yields a machine efficiency map peaking at 86.3% and a sampling-rate bound. Subsampled without filtering, cycle energy stays within 0.14% down to 2 kHz at every sampling phase, low enough to make an electrical channel beside the real-time control link worth testing. These figures are established on one drive unit, with the fidelity repeats taken in a single session; unit-to-unit variation and day-to-day reproducibility are the subject of further work.

The natural next step is validation-oriented work for software-defined drives: using a bench characterized this way to decide whether a controller update shipped to a fleet has altered the drive’s energy behavior by more than the platform’s own resolution. That question needs the repeatability figures established here as its scale, and it needs energy measured under the emulated load, which a low-rate electrical channel on the real-time path could provide. Both are the subject of ongoing work.

## Figures and Tables

**Table 1:** Measured platform characteristics and fitted models.

Item	Value	Source
Drive under test		
Speed per volt of throttle	1.783 (m/s)/V	measured
Torque ceiling model	$4.64\;N\cdot m/V\;V_{\mathrm{th}}-0.64\;N\cdot m$	fitted at 1.0 and 2.0 V
Ceiling cross-check at 2.5 V	10.96 N·m predicted, 10.78–10.98 N·m measured	measured, three days
Switching frequency	26 kHz	measured, phase-voltage spectrum
Load unit		
Calibrated static curve	to 50% of command, 11.8 N·m at the top	measured, lookup
Command deadband	4.75%	measured
Minor-loop hysteresis	0.16 N·m	measured
Dead time/lag	0.36 s/0.16 s	measured, 96 edges
Bench mechanics		
Rotating inertia	0.0087 kg m^2^ (0.54 kg equivalent)	measured, coastdown
Real-time loop		
Measurement link	100 Hz nominal, 99.8 Hz achieved	measured

**Table 2:** Measurement channels. All analog inputs are acquired synchronously at 200 kS/s; derived and encoder-based channels are published asynchronously. Noise floors σ are measured at full bandwidth with the bench at rest, except the speed channel, which quantizes to zero at standstill and was measured at a steady 250 rpm instead (Section 5).

Channel	Quantity	Range	Acquisition	Noise Floor σ
Torque	shaft torque	±30 N·m	synchronous, 24-bit	0.00868 N·m
Tachometer	pulse train	—	synchronous, 360 pulses/rev	—
Speed	road speed	—	derived, ∼500 Hz	0.113 km/h
Voltage phase 1–3	machine terminal	—	synchronous	0.044 V–0.058 V
Current phase 1–3	machine terminal	60 A range	synchronous	0.121 A–0.130 A
Input voltage	DC bus	—	synchronous	0.0468 V
$P_{L1..L3}$, *P*	active power	—	derived	—
$P_{\mathrm{mech}}$	mechanical power	—	derived from torque ×ω	—

**Table 3:** Emulation accuracy over the scaled ECE-15 cycle, mean [95% confidence interval] with the coefficient of variation (CoV) of the warm subset. The pooled column is all eight runs; the warm column excludes the cold first run of the session and is reported as a post hoc sensitivity analysis, not as the headline. All intervals are descriptive summaries of repeats taken in one session (Section 5.1). Averaged over the runs, the peak measured speed was 25.41 km/h against the 25.0 km/h reference; no run reached the output rail and no stall protection trip occurred.

Metric	Pooled (n=8)	Warm (n=7)	CoV, Warm
Command path (cap retention)			
Impulse delivered	99.51% [99.05, 99.97]	99.71% [99.70, 99.72]	0.01%
Cap impulse loss	0.49% [0.03, 0.95]	0.29% [0.28, 0.30]	4.61%
Cap active time	8.38% [7.50, 9.27]	8.02% [7.76, 8.29]	3.57%
Measured path (transducer and speed channel)			
Measured impulse	103.16% [100.56, 105.76]	102.06% [101.81, 102.32]	0.27%
Torque RMSE vs. setpoint	0.704 N·m [0.514, 0.893]	0.623 N·m [0.616, 0.630]	1.22%
Torque RMSE vs. post-cap command	0.700 N·m [0.511, 0.889]	0.620 N·m [0.613, 0.627]	1.22%
Speed tracking RMSE	0.593 km/h [0.375, 0.811]	0.501 km/h [0.487, 0.515]	2.92%

## Data Availability

The analysis scripts and computed result files from which every figure reported here is generated are available from the corresponding author on reasonable request. The raw full-rate acquisition files are retained locally on account of their size.

## Abbreviations

The following abbreviations are used in this manuscript:

AC	alternating current
CI	confidence interval
CoV	coefficient of variation
DC	direct current
ECE	Economic Commission for Europe (drive cycle family)
PI	proportional–integral
RMSE	root-mean-square error
RQ	research question
UDP	User Datagram Protocol
